# N-P Fertilization Stimulates Anaerobic Selenium Reduction in an End-Pit Lake

**DOI:** 10.1038/s41598-017-11095-2

**Published:** 2017-09-05

**Authors:** Andreas Luek, David J. Rowan, Joseph B. Rasmussen

**Affiliations:** 1 Department of Biological Sciences, University of Lethbridge, 4401 University Drive, Lethbridge, T1K 3M4 Alberta Canada; 2 Environmental Science and Monitoring Branch, Canadian Nuclear Laboratories, 286 Plant Road, Chalk River, K0J 1J0 Ontario Canada

## Abstract

Selenium (Se), an essential micro nutrient, is toxic to aquatic life at slightly higher water concentrations. Watersheds receiving leachate from selenium rich sources require large-scale, long-term treatment to mitigate Se toxicity. We applied the principles of anaerobic bacterial bioreactors, previously successful in small scale Se mitigation, to a whole end-pit lake ecosystem. Fertilization of the lake with N and P increased primary production, creating a meromictic, anoxic layer, and enhanced the habitat for locally present, anaerobic, Se and sulfur reducing bacteria. Within two years, Se concentrations were reduced ten-fold, reaching water-quality guideline values. The successful experiment demonstrated a novel treatment of large volumes of Se-contaminated water, and introduced an inexpensive method to mitigate a persistent aquatic pollutant of global concern.

## Introduction

Selenium (Se) contamination of aquatic environments is predominantly caused by anthropogenic activity, and is a contaminant of global concern^1^. Se cycles between the geosphere and the hydrosphere naturally^2, 3^; however, industrial activities such as mining and combustion of coal, mining and smelting of metal ores, agricultural irrigation of seleniferous soil, as well as certain industrial manufacturing processes, introduce Se more rapidly to aquatic environments than it is removed by natural processes^2, 4^. As a trace element, selenium is an essential nutrient, that, at even marginally higher concentrations, can be highly toxic to aquatic life, often resulting in teratogenic effects in fish and other egg laying vertebrates^5^. The most common dissolved forms of Se, or those available for uptake by biota, are the Se oxyanions selenate (SeO_4_^2−^, Se VI) and selenite (SeO_3_^2−^, Se IV), which require chemical reduction processes to be converted into less soluble, biologically inert, elemental selenium (Se°)^1^. Hence, Se oxyanions remain persistent in the environment for a long time, unless actively reduced by physical, chemical or biological treatment methods^2^. The challenge of transferring successful bench scale size experiments to large scale applications^6, 7^, as well as high costs of treatment methods, have so far limited large scale remediation successes^1^, which is why Se pollution remains a significant environmental issue worldwide^1, 2^.

Active and passive reduction processes to mitigate aquatic Se include direct chemical treatment^2^, reverse osmosis^2^, and biological treatment options, such as artificial wetlands^8, 9^ and bioreactors^7^. Bioreactors are a common tool for biological treatment of wastewater^10^, and this method has been successfully used to reduce the concentration of Se in coalmine effluent on a pilot-scale^7, 11^. Experimental Se reducing bioreactor applications utilize microbial communities, that metabolize selenate (Se VI) and selenite (Se IV) to elemental Se (Se°) under anaerobic conditions^7, 12^. Communities of anaerobic Se reducing bacteria (SeRB) can consist of obligate Se VI and Se IV reducers^12^, as well as sulfate reducing bacteria (SRB), capable of using both Se and sulfate as their terminal electron acceptor^13^. In bench scale experiments, specific strains of bacteria have been shown to successfully reduce Se oxyanions under very controlled environments^14–17^, while small-scale mesocosm experiments utilized unidentified bacterial communities for successful treatment of small volumes of Se-rich water^7, 18^.

In a bioreactor, the required environment for anaerobic respiration and reduction of Se can be highly controlled and optimized for the quickest and most efficient removal of the contaminant^2^. However, the high level of control required and the associated costs are the biggest obstacles hindering large scale industrial application^2^.

In principle, a bioreactor treating Se-contamination requires i) organic matter as substrate, in ii), an anaerobic environment that encourages the proliferation of iii), a bacterial community capable of reducing selenates and selenites by anaerobic respiratory processes^7^. In the absence of oxygen, anaerobic respiration pathways use other oxyanions as electron acceptors, such as nitrate, nitrite, selenium oxyanions and sulfates^2^. The energetic benefit of each electron acceptor depends on its redox potential, and the reduction process will be inhibited by an electron acceptor with a higher redox potential^12^. O_2_ has the highest redox potential (E_0_’ = 0.81 V) followed in descending order by nitrate (NO_3_^−^/N_2_; E_0_’ = 0.75 V), selenium oxyanions (Se^VI^: SeO_4_^2−^/SeO_3_^2−^; E_0_’ = 0.48 V, Se^IV^: SeO_3_^2−^/Se_0_; E_0_’ = 0.21 V) and sulfate (SO_4_^2−^/SO_3_^2−^; E_0_’ = −0.516 V)^12^. In result, Se reduction has to proceed from Se^VI^ to Se^IV^ to Se^0^. Although both steps of this reduction process are energetically less beneficial than the reduction of nitrate, they will precede the reduction of sulfate^12^.

The mining and combustion of coal is one of the largest industrial vectors of Se release to the environment globally^1^. Even though a declining market for coal, and phasing out coal fired power plants, has forced a large number of open pit mines to close across the world^19, 20^, a legacy of disturbed landscapes and watersheds with a need for reclamation is left behind in the environment^21^. A large part of the environmental legacies of open pit mining are end-pit lakes. Pit lakes are created as a reclamation tool for surface mine excavations, by backfilling the excavated pit with water from the surrounding watershed^22^. The morphometry of pit lakes follows that of the excavated pit, often with characteristically high ratios of water depth to surface area as well as long water residence times^23^.

Water quality in coalmining pit lakes is a function of the surrounding geology^24^, and ionic composition is strongly dominated by leachates from mining activity^24^. Large pyrite deposits can lead to acid mine drainage, especially in areas high in sulfides and low carbonates, while gypsum is calcium rich and buffers drainage water to a circum-neutral to alkaline pH^24^. As a persistent contaminant often associated with coal mining activities, Se is leached from waste rock. Se co-occurs with sulfates, which are usually present in large excess of Se, but with significantly less threat to the aquatic environment^23, 25^.

The small surface area of an end-pit lake limits wind mixing and increases the likelihood of permanent stratification, or meromixis^26^. Meromictic lakes are characterized by a chemocline, which prevents the monimolimnion from exchanging gasses with the atmosphere^26^. The epilimnion in a meromictic lake can effectively isolate hypolimnetic water from the surrounding environment, trapping contaminants of concern in this monimolimnion^27, 28^.

We suggest a treatment of Se contaminated water, in which the principles of a bioreactor are applied *in-situ* to stimulate anaerobic reduction processes utilizing the hypolimnion of end-pit mine lakes. Following the principles of a bioreactor, we hypothesize, that a nutrient amendment to an end-pit lake will, (i) increase primary production, (ii) reduce dissolved oxygen in the hypolimnion and aid the development of a biogenic, meromictic lake^26^, and (iii) promote the growth of an anaerobic SeRB community in the monimolimnion that will reduce the Se concentration in the water.

Nutrient amendments have shown to dramatically increase primary production in oligotrophic lakes^29^, while sinking, decomposing organic matter has been identified as a major cause for anoxic hypolimnia in eutrophic lakes^30^. While a phytoplankton bloom might not be possible in acidic mine-pit lakes, neutral to alkaline pit-lakes should respond positively to nutrient additions.

SeRB and SRB are ubiquitous in the environment^12^, naturally present in lake sediments below the oxygen horizon^13^, and have been found in coal mining pit lakes^7^. An inoculation with a specific, known bacterial community is therefore unnecessary. We predicted that an increase in resources and the expansion of anoxic habitat will increase SeRB abundance, expand SeRB habitat from the sediment into the anoxic water column and subsequently reduce Se in the newly established monimolimnion.

We tested our hypotheses on a moderately sized, accessible end-pit lake of roughly 7 ha area and 45 m maximum depth on an abandoned mine site near Grande Cache, Alberta, Canada. Se concentration of the lake before the experiment was 6.5 µg/L throughout the water column, well above the 1 µg/L Alberta provincial, and Canadian federal, water quality guideline value^31, 32^. Selenate (Se VI) was the most abundant Se oxyanion (66.8%), followed by selenite (Se IV) at 13.1%, in the pit-lake water.

In May 2012, 15 t of a N and P mix fertilizer (10% N; 34% P by weight as ammonium polyphosphate) were added to the lake, followed by an additional fertilization of 40 t of N fertilizer (28% N, 0% P) in August 2013. Commercial, liquid, agricultural fertilizer was used for both additions. The two nutrient amendments raised nitrate from 0.1 mg/L to 0.6 mg/L at the surface and 3.8 mg/L at the bottom of the lake. Phosphorus increased from 0.001 mg/L to 1 mg/L at the surface and 1.3 mg/L at the bottom after fertilization.

We monitored the experimental lake from a month before the first fertilization in June 2012 until the summer of 2016, three years after the fertilization. We measured vertical lake profiles of temperature and dissolved oxygen at one meter intervals, measured Secchi depth, and took water samples for a suite of water quality variables, including nitrate, selenium and sulfate at two depths (top: 0.5 m below surface, bottom: ~2 m above lake bottom). Sampling took place approximately once a month during the ice-free season as well as once a year under the ice, weather permitting.

**Figure 1:**
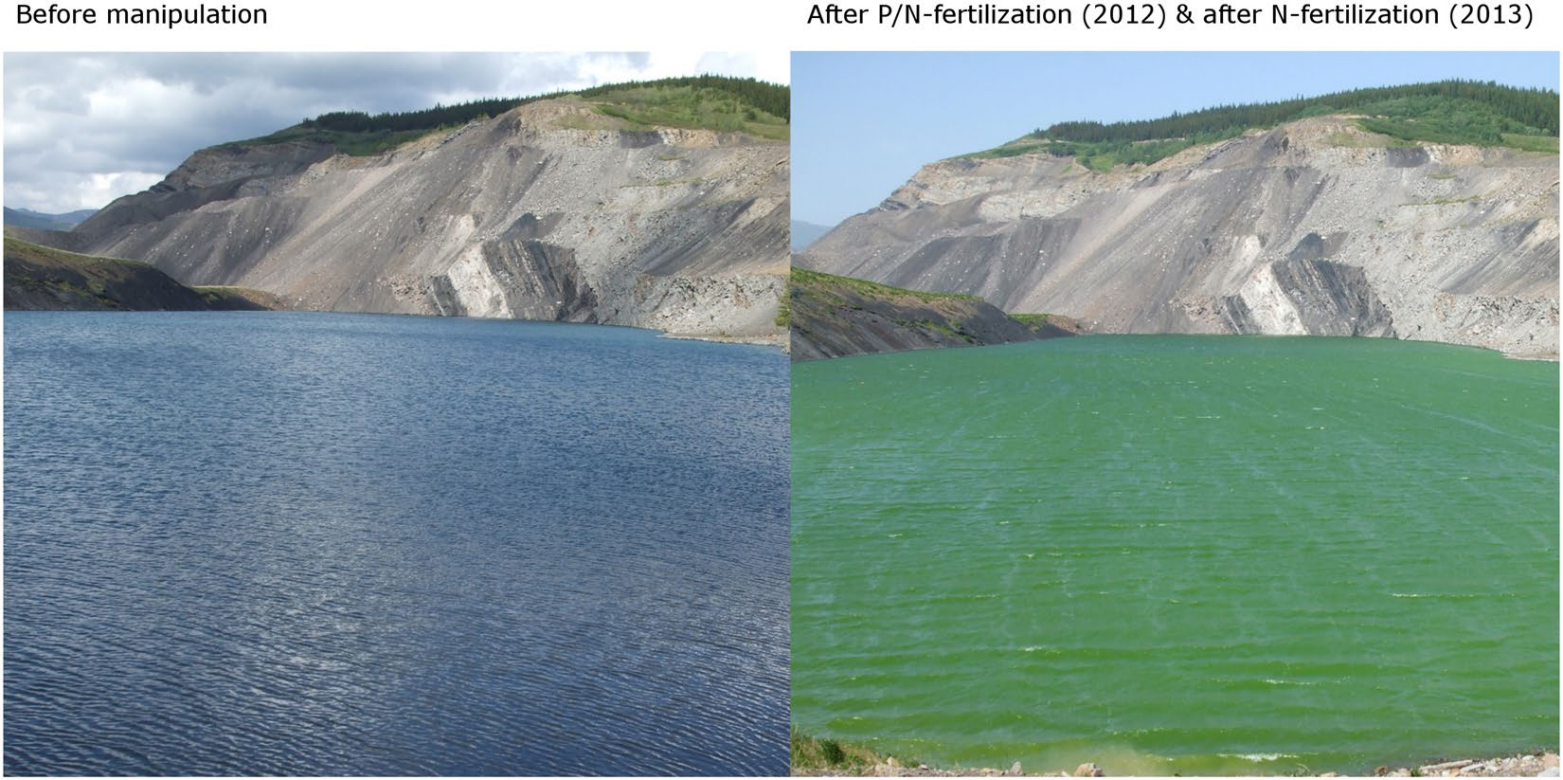
Picture of the manipulated lake before and after nutrient amendments.

**Figure 2:**
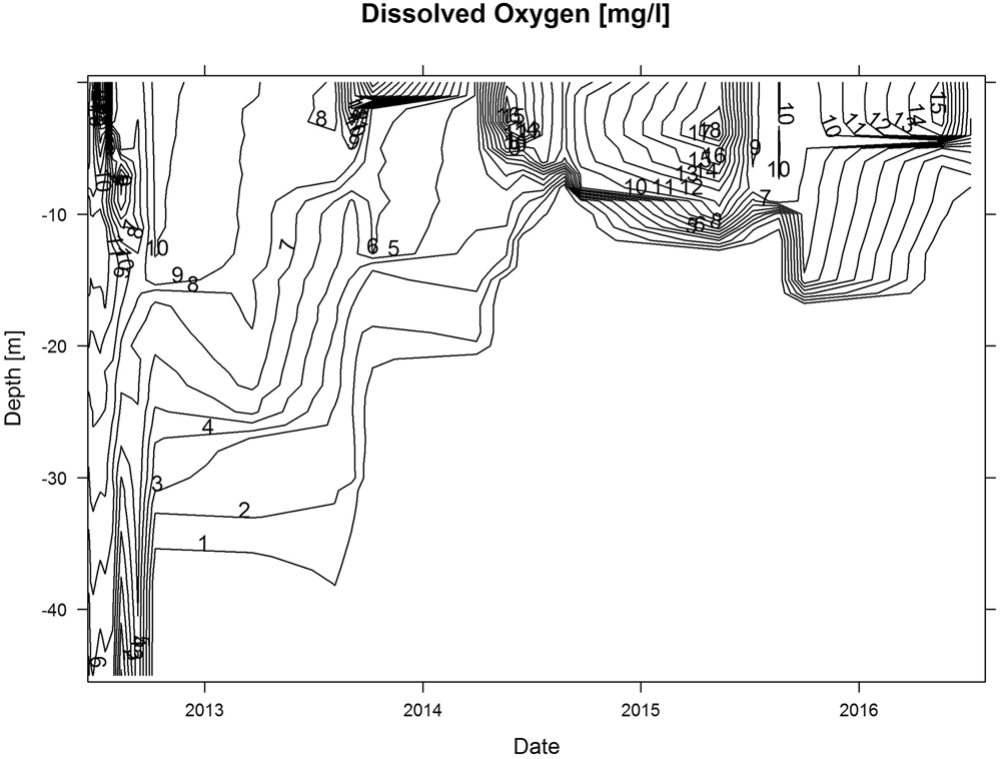
Dissolved oxygen profile of the experimental lake over time. Lines depict 1 mg/L increments.

Phytoplankton began to bloom shortly after the first nutrient amendment (Fig. 1). This bloom lasted for two weeks and depleted nitrate at the surface. After the second fertilization, phytoplankton blooms persisted throughout the ice free season. The first phytoplankton bloom was dominated by *Chlorella* spp., a single celled green alga. After the second fertilization, the initial bloom was predominantly *Synechococcus* spp., which later shifted towards a diatom community, as evidenced by brown-green water color and depletion of dissolved silica in the surface water samples (see Supplemental Material/Figure A1). Phytoplankton was abundant during the remainder of the experiment. Secchi depth was never more than 2 m after the first bloom, compared to 9 m before the manipulation. Surface nutrient concentrations declined over the course of each year. While the lake thermally stratified in the summer, winter water temperatures were not stratified and allowed for diffusion of nutrients from deeper water depths. However, the anoxia in the monimolimnion was maintained (Fig. 2).

Surface nutrient concentrations varied by season and Se and sulfate concentrations fluctuated, possibly in response to variations in precipitation. However, the size of the lake buffered these variations in the bottom samples. Furthermore, due to the isolation of the anoxic monimolimnion from the surface water of the manipulated lake, effects of the surrounding environment would likely be limited to the surface layer of the lake, while the experimentally induced response is hypothesized to happen primarily in the hypolimnion. The focus of the following discussion will hence be on the changes of hypolimnetic water quality.

Phase I, the aerobic phase, begins with a sustained algae bloom initiated with the first fertilizer amendment (supports step 1; increased primary production), and concludes with the subsequent establishment of a stable, anoxic, biogenic, hypolimnion (step 2; decomposition of organic matter depletes oxygen). In Phase II, intermediary electron acceptors, especially nitrate left over from the fertilization, were reduced anaerobically, before the anaerobic respiration of selenate and selenite in phase III (step 3; microbial reduction of Se).

**Figure 3:**
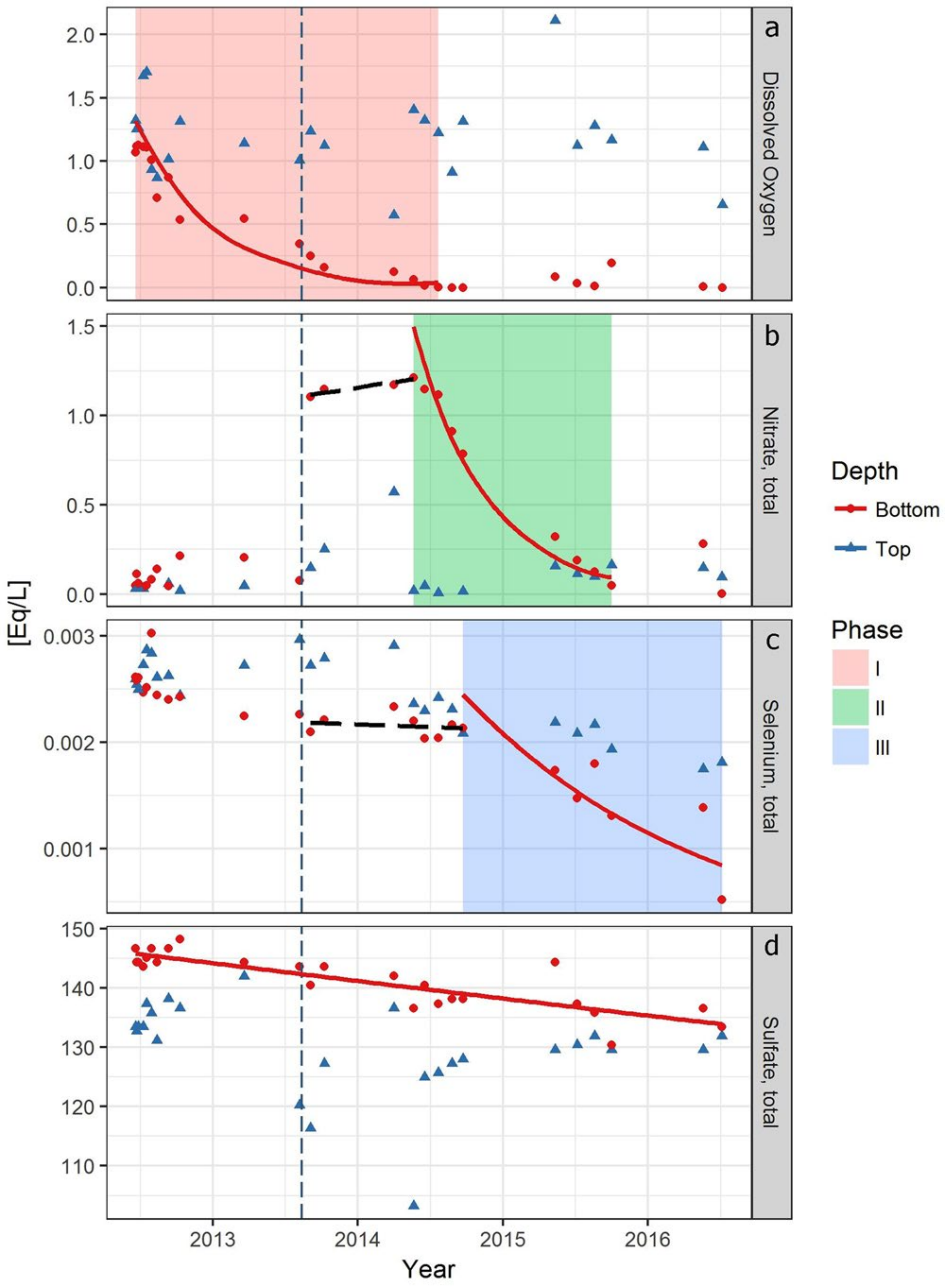
Graphs of oxygen (**a**), nitrate (**b**), selenium (**c**) and sulfate (**d**) electron equivalences per liter over time. Blue triangles represent values for surface samples, red circles represent bottom samples. Solid red lines are significant regressions (p < 0.05) of natural log transformed values of the reduction of each electron acceptor over the indicated time, representative of the timing of the element’s reduction by biological processes. Black dashed lines are non-significant regressions for nitrate and selenium before the onset of the element’s reduction. Dashed vertical dark blue line marks time of final fertilizer addition. Shaded boxes mark the phases of electron acceptor reductions.

To analyze reduction processes in the hypolimnion, concentrations of electron acceptors were expressed as electron equivalencies (e.g. Eq/L) and followed over time (Fig. 3). Over the course of the experiment, oxygen, nitrate and Se concentrations declined in that order in the hypolimnion, as expected from their redox potentials^12^. Reduction of the electron acceptors followed first order kinetics (y_t_ = y_0_ * e^−kt^), by which the slope of the log transformed equivalency concentrations over time are a representation of the concentration change per unit time (t) i.e. the decay constant k^33^.

Phase I (aerobic phase) lasted from the first fertilizer treatment to the depletion of hypolimnetic oxygen in June 2014, where oxygen was reduced at a yearly decay constant of k_O2_ = 1.9 yr^−1^. In phase II, nitrate was depleted from the water column (June 2014 to July 2015) at a yearly rate of k_NO3_ = 2.1 yr^−1^. Following nitrate depletion, phase III saw the depletion of Se from the water column (September 2014 to June 2016) at a much lower yearly decay constant of k_Se_ = 0.6 yr^−1^ (Table 1).

**Table 1:** Phases of the reduction process in the manipulated lake.

Electron acceptor	Phase	Start	End	p-value	R^2^	decay [Eq/L * y]
Oxygen	I	June 2012	July 2014	<0.0001	0.83	1.9
Nitrate	II	May 2014	October 2015	<0.0001	0.92	2.1
Selenium	III	September 2014	June 2016	0.036	0.54	0.6
Sulfate		June 2012	June 2016	<0.0001	0.71	0.02

Regression results of the decay of each electron acceptor were calculated from data within the timespan of each phase. Data was natural-log transformed. The decay was calculated per day and transformed to per year.

In Phase I, the decomposition of sinking algae eliminated oxygen from the hypolimnion in less than two years. The first phytoplankton bloom in 2013 initiated the reduction of oxygen in the hypolimnion. An anoxic layer established during the winter of 2013–2014 which increased in size from the bottom up in 2014, until establishing a stable, anoxic zone below 10 m water depth in August 2014 (Fig. 2 and Fig. 3a). Continued decomposition of organic matter maintained anoxia in the hypolimnion to date and, without any seasonal vertical mixing with the epilimnion, a biogenic monimolimnion established in the lake.

The nutrient addition to increase primary production also added nitrate to the hypolimnion. Denitrification in Phase II began, once oxygen was completely eliminated from the hypolimnion after Phase I (Fig. 3b). Nitrate concentrations stabilized below 1 mg/L (<0.1 Eq/L) by July 2015 and remained stable below this concentration after this time.

Se concentrations remained unchanged after fertilization until nitrate dropped below 0.3 Eq/L. Total Se concentrations in the water column began to decline sharply over the winter of 2014–2015 (Phase III) and approached the CCME water quality guideline values of 1 µg/L at the last measurement in June 2016. The decay constant for Se of 0.6 yr^−1^ is lower than oxygen or nitrate, likely due to the increased number of steps for the reduction process from selenate, to selenite to Se^0^ as well as the lesser energy return of the redox process.

Sulfate has the lowest redox potential of the abundant elements in the pit lakes. Initial sulfate concentrations exceeded nitrate by 10^2^, and Se by 10^6^. A steady monotonic decline of sulfate was observed, however, reduction was less than 5% with a decay constant of k_S_ = 0.02 yr^−1^ over the course of the experiment.

The reduction of oxygen, nitrate and Se in the monimolimnion reflects microbial metabolic activity. Distinct groups of bacteria and archaea are capable of utilizing nitrate, Se-oxyanions, and sulfate as electron acceptors in respiratory processes in the absence of oxygen^12^. Nitrate reducing bacteria only use nitrate as an electron acceptor^34^, while there is also evidence of Se reducing bacteria using nitrate and selenate as their terminal electron acceptor^18, 35^. Selenate and selenite can be reduced by Se- and sulfur reducing bacteria alike^2, 12^.

Our experiment showed a sequential reduction of nitrate before Se, following the chemical redox potential from high to low, i.e. nitrate to selenium^2, 12^, with some overlap between Phase II and III (Fig. 3). One would assume, that nitrate should be completely eliminated, before Se reduction can commence. However, in the experimental lake, Se concentrations started to decline before nitrate was completely eliminated from the system. In high concentrations, nitrate inhibits the microbial reduction of selenate^34^, and favors the reduction of nitrate. In lower nitrate concentrations, selenate reduction can commence in the presence of nitrate^18, 34^, and a SeRB community can compete and coexist with denitrifiers. Oremland *et al*.^18^ showed in a lab test, that at Se and nitrate concentrations similar to this field experiment, Se was reduced by *Sulforospirillum barnesii* at ambient millimolar Se concentrations, while nitrate was present at a 1000x molar excess. In an environment, where electron donors are not limiting due to permanent input of decomposing organic matter to the monimolimnion, the reduction of nitrate and selenate can occur simultaneously^18^. Equivalency values of nitrate in the lake were only at 100x excess of total Se equivalencies, when reduction of Se commenced. We suggest that when nitrate concentrations fell below 1 mg/L in June 2015, the continued input of carbon from the sinking, decaying algae as the electron donor exceeded the biomass needed by the nitrate reducing bacteria. The excess organic matter allowed the SeRB community to grow and reduce Se-oxyanions in the presence of nitrate. Furthermore, it is possible for some bacteria such as *Thaurea selenatis*, to reduce selenate together with nitrate by a metabolic pathway, in which nitrite reduction catalyzes the final step for the reduction from selenite to elemental selenium^35^.

The dissimilatory reduction of Se can be achieved by obligate SeRB and SRB^12^. We did not identify specific bacterial strains, however, both SeRB and SRB are ubiquitous in the environment^12, 13, 36^ and were previously found in our experimental lake system^7^. While Se respiring bacteria are specific to only Se oxyanions, a variety of sulfur respiring bacterial will preferably metabolize selenate before sulfate, due to its structural similarity with higher energy return^18, 37^. This preferential use of Se oxyanions is even achieved in a large excess of sulfur^12^. In our experimental lake, sulfate equivalencies were six orders of magnitude higher than Se, yet Se was reduced, while sulfur concentrations did not show a response to the manipulation. While the reduction of nitrate and Se is likely achieved by two different groups of bacteria, the reduction of Se and sulfate is more likely to be achieve by the same group of bacteria^12, 13^. Hence, the reduction of any Se oxyanions has to be complete, before sulfate can be reduced at increased amounts.

We were able to show, that it is possible to reduce selenium concentrations in an end-pit lake using ecosystem manipulation. We combined the knowledge of existing bioreactor principles with the *in-situ* production of a carbon source within the lake, rather than a direct addition of organic carbon such as whey or molasses. The mechanisms of microbial Se reduction processes were already well understood, but the limitation to successfully treat a whole lake ecosystem was hindered in large parts by the need for enough energy in form of a carbon source^38^. Attempts have been made before to introduce a carbon source and prime a lake with the required bacterial community^39–42^, but the amount of organic matter required makes application in larger lakes not feasible^38^. By adding nutrients, the yield of captured carbon through primary production is orders of magnitude higher and requires only a fraction of the volume to be brought to the lake, making it logistically much more feasible.

A careful control of the nutrient balance should accelerate the process of Se reduction. If added nitrate can be prevented from sinking into the hypolimnion before being assimilated, the Se reduction phase will be less delayed. We estimate that the onset of Se reduction would have occurred 1.5 years earlier if nitrate had been retained in the epilimnion. A more precise stoichiometric approach to the nutrient addition would have required only one amendment, which would reduce the response time by another year. Thus, we predict, that the addition of precise volumes of fertilizer to the surface of an end-pit lake can show first results shortly after oxygen is depleted in the hypolimnion and a measurable response of Se reduction should be possible within the second growing season after the nutrient amendment.

To further improve water quality of mine discharge, rerouting of smaller streams through the monimolimnion of a fertilized pit-lake may allow the transformed pit lake to be used as a flow-through bioreactor itself. Additional monitoring of the monimolimnion and water inflow would be required to ensure that anoxia is maintained. A continuous supply of nutrients would also be required to maintain the input of organic material to the hypolimnion. The treatment of flowing water in an end-pit lake would negate the need to build physical structures for bioreactors and reduce the costs of mitigation from millions to thousands of dollars. This method would also address the problem of re-contamination of treated water, when water discharging from a pit-lake flows through subsurface rock drains.

Treating Se contamination by ecosystem manipulation is a method best suited for large and deep lakes with small surface area to volume ratios and circum neutral pH. We propose the use of this method in lakes with longer water residence times (>12month) and maximum depths significantly deeper than their euphotic zone. There are in excess of 40 coal-mining end-pit lakes larger than one hectare in Alberta, Canada alone. From the pit-lakes we were able to obtain basic data for, 61% (22 out of 36 lakes) were larger than 2 ha and had a relative depth larger than 10 with a pH between 6.7 and 8.6. This combination of relative depth and size of the lake insures, as a rough estimate, that they are deep enough to stratify and have steep enough slopes to promote meromixis. Hence more than half of the coal-mining pit lakes in Alberta are suitable for the herein described method of Se treatment. With large numbers of coal mines predicted to close in the near future, the amount of pit lakes in the landscape will increase making inexpensive and efficient treatments of critical importance. Since all of these lakes require continuous water quality monitoring mandated by the rules for mine closure operations, the additional cost of the fertilizer is essentially minimal. We believe, that by adapting the herein described method to other industries, the concept offers an inexpensive adaptation and large scale application for the mitigation of aquatic pollutants that require chemical reduction processes in anoxic environments.

## Methods

### Study area

The study lake used in this experiment is located in the eastern rocky mountain foothills north of Grande Cache, Alberta Canada (UTM 5989903.74 N, 356179.51E). The experimental lake is 7.25 ha in surface area with a maximum depth of 45 m. The experimental lake does not have a surface outflow. A subsurface rock drain with a flow of <1 m^3^/s gives the lake a water retention time of >1 year. A summary of water chemistry can be found in the supplementary material (table S1).

A smaller upstream lake, connected by a small surface connection, was initially considered as a reference lake. It was however deemed not suitable as a directly comparable lake due to its small surface area of 2.45 ha and shallow maximum depth of 12 m. While monitoring commenced on both lakes with equal effort, the data on the smaller lake revealed very high variability on all water quality metrics due to continuous mixing, a lack of stratification, and strong influences of weather, especially precipitation events. We decided that any effects of the manipulation in the manipulated lake would be clearly identifiable and possible to separate from natural variability. Hence, the upstream lake is not included in the analysis and data is not shown in this document. Both lakes are abandoned surface mine pits that were filled with water from the surrounding landscape as a remediation tool and remain fishless.

### Lake fertilization

The manipulated lake was fertilized in May 2012 and August 2013. No long term data was available prior to fertilization. As a highly oligotrophic headwater pit-lake, we assumed phosphorus to be limiting with negligible concentrations and nitrogen to be potentially higher due to input from blasting operations during mining^43, 44^. We estimated the volume of the lake from its surface area and depth and chose a target concentration for total P as roughly 0.5 mg/l. In the first fertilizer amendment, 15 t of a P-heavy liquid ammonium phosphate agricultural fertilizer (10% N; 34% P by weight) was added to the lake within two weeks. Half of the fertilizer was added from shore in one dump, the other half was stored in tanks on shore and added over two weeks in the propeller-wash of a boat to better mix the fertilizer at the surface. Total P concentrations spiked immediately after fertilization and settled to 1 mg/l at the surface and 1.3 mg/L at the bottom of the lake after two months.

A second fertilizer amendment was necessary, after it was evident, that the low concentration of N in the lake was insufficient to sustain a phytoplankton bloom for more than one month. The second fertilization event in August 2013 added 40 t of an agricultural, commercially available, liquid nitrogen fertilizer (28% N, 0% P) to the manipulated lake, which contained a mix of 50% nitrogen from urea, 25% nitrogen from ammonia and 25% nitrogen from nitrate. This time, the fertilizer was directly discharged into the lake from shore within 30 min. Dispersion of the fertilizer within the lake happened over a few weeks and raised the lakes nitrate concentration from 0.1 mg/L to 0.6 mg/l at the surface and 3.8 mg/L at the bottom. The high specific gravity of the fertilizer caused it to accumulate in higher concentrations in the deeper parts of the lake.

### Water quality analysis

Samples were collected three times within one month before the first fertilization, and monthly during the ice free season after that. Additionally, water samples were taken once during the winter from under the ice, if conditions allowed for it. The lake was inaccessible during the winters of 2014–2015 and 2015–2016, due to unstable ice and extremely bad road conditions on unmaintained abandoned mine roads. Water quality samples and depth profiles were taken over the deepest spot of the lake. Water temperature and dissolved oxygen were measured as depth profiles at one meter increments with a Hydrolab DS5 multiprobe outfitted with a HACH LDO sensor for dissolved oxygen connected to a Surveyor 4a handheld data recorder (HACH Environmental, Colorado, USA).

Water quality samples were taken at two depths, over the deep spot of the lake, using a Van-Dorn water sampler. Surface samples were taken at 0.5 m below the surface, while bottom samples were taken between 1–2 m above ground. Samples were stored in acid washed bottles, preserved in the field with trace metal grade acids and kept refrigerated until analysis. Samples were analyzed for standard ion composition, nutrients, and metals by ALS Environmental in Edmonton, AB to laboratory standards, according to the Canadian Association for Laboratory Accreditation requirements ISO/IEC 17025^45^. Due to costs, only in 2014, water was additionally analyzed for Se speciation at the Water Quality Center at Trent University in Peterborough, ON, Canada. Samples were stored in acid washed bottles and shipped over night in coolers to maintain a preserving environment. Selenium speciation was analyzed by anion exchange chromatography-hydride generation-inductively-coupled plasma-dynamic reaction cell-mass spectrometry (AEC-ICP-DRC-MS) following the methods outlined in Wallschläger and Roehl^46^ and Wallschläger and London^47^.

### Data analysis

Data were statistically analyzed using the programming language R^48^. Graphical representations of the data were created using the libraries ggplot2^49^ and lattice^50^. Water quality data was graphed over time. The three phases of the reduction process were identified a-priory. Timing of each phase was set as the change in slope of the element at the beginning of the phase and the stabilization of a low point at the end of the phase. Their time intervals were marked as Phase I for oxygen reduction, Phase II for nitrate reduction, and Phase III for selenium reduction (Table 1, Fig. 3). The change of concentrations of each element was expressed as electron equivalencies, i.e. the amount of electrons, that were taken up by each electron acceptor per mol of the element. We used an electron transfer of four electrons for oxygen, five electrons for Nitrate and eight electrons for sulfate. In the case of Se reduction, we used the ratio of the Se-oxyanions measured in 2014 to calculate Se electron equivalencies more precisely. We used the total Se concentrations and calculated the ratio of electrons transferred from the ratio of Se-oxyanions measured in 2014. The reduction of Selenate (SeVI) takes six electrons, while the reduction of selenite (SeIV) only takes four electrons.

We assumed a first order relationship for the reduction of electron acceptors^33^, i.e. y_t_ = y_0_ * e^−kt^, where y_0_ is the start concentration, k is the decay constant, and y_t_ is the predicted concentration of the electron acceptor at time t. Hence, the reduction of each electron acceptor was compared over time using linear regression models for each phase separately (Table 1). The slope of each regression represents the first order decay constant k (yr^−1^).

## Electronic supplementary material

Supplementary Information

## References

[1] Lemly AD (2004). Aquatic selenium pollution is a global environmental safety issue. Ecotoxicol. Environ. Saf..

[2] Lenz M, Lens PNL (2009). The essential toxin: the changing perception of selenium in environmental sciences. Sci. Total Environ..

[3] Fernández-Martínez A, Charlet L (2009). Selenium environmental cycling and bioavailability: a structural chemist point of view. Rev. Environ. Sci. Biotechnol..

[4] Hopkins RL, Altier BM, Haselman D, Merry AD, White JJ (2013). Exploring the legacy effects of surface coal mining on stream chemistry. Hydrobiologia.

[5] Lemly AD (1993). Teratogenic effects of selenium in natural populations of freshwater fish. Ecotoxicol. Environ. Saf..

[6] Cantafio AW (1996). Pilot-Scale selenium bioremediation of San Joaquin drainage water with Thauera selenatis. Appl. Environ. Microbiol..

[7] Luek A, Brock C, Rowan DJ, Rasmussen JB (2014). A simplified anaerobic bioreactor for the treatment of selenium-laden discharges from non-acidic, end-pit lakes. Mine Water Environ..

[8] Ye ZH, Lin Z-Q, Whiting SN, de Souza MP, Terry N (2003). Possible use of constructed wetland to remove selenocyanate, arsenic, and boron from electric utility wastewater. Chemosphere.

[9] Schmidt R (2013). Selenium biotransformations in an engineered aquatic ecosystem for bioremediation of agricultural wastewater via brine shrimp production. Environ. Sci. Technol..

[10] Grady, L. C. P., Daigger, G. T., Love, N. G. & Filipe, C. D. M. *Biological wastewater treatment*. (CRC Press Taylor & Francis Group, 2011).

[11] Lenz M (2008). Selenate removal in methanogenic and sulfate-reducing upflow anaerobic sludge bed reactors. Water Res..

[12] Nancharaiah YV, Lens PNL (2015). Ecology and biotechnology of selenium-respiring bacteria. Microbiol. Mol. Biol. Rev..

[13] Muyzer G, Stams AJM (2008). The ecology and biotechnology of sulphate-reducing bacteria. Nat. Rev. Microbiol..

[14] Hageman SPW, van der Weijden RD, Weijma J, Buisman CJN (2013). Microbiological selenate to selenite conversion for selenium removal. Water Res..

[15] Oremland RS (1994). Isolation, growth, and metabolism of an obligately anaerobic, selenate-respiring bacterium, strain SES-3. Appl. Environ. Microbiol..

[16] Macy JM, Lawson S, Demoll-Decker H (1993). Bioremediation of selenium oxyanions in San Joaquin drainage water using Thaurea selenatis in a biological reactor system. Appl. Microbiol. Biotechnol..

[17] Hunter WJ, Manter DK (2009). Reduction of selenite to elemental red selenium by Pseudomonas sp. Strain CA5. Curr. Microbiol..

[18] Oremland R, Blum J (1999). Simultaneous reduction of nitrate and selenate by cell suspensions of selenium-respiring bacteria. Appl. Environ. Microbiol..

[19] Davenport, C. U. S. pledges to ease pain of closing coal mines in shift to cleaner energy. *The New York Times* 16–19 (2017).

[20] Jamasmie, C. China shutting another 4,300 coal mines. *Mining.com* Available at: http://www.mining.com/china-shutting-another-4300-coal-mines/ (2017).

[21] Hildmann E, Wünsche M (1996). Lignite mining and its after-effects on the Central German landscape. Minesite Recultiv..

[22] Castro JM, Moore JN (2000). Pit lakes: their characteristics and the potential for their remediation. Environ. Geol..

[23] Gammons, C. H., Harris, L. N., Castro, J. M., Cott, P. A. & Hanna, B. W. *Creating lakes from open pit mines: processes and considerations, with emphasis on northern environments- Canadian Technical Report of Fisheries and Aquatic Sciences 2826*. *Fisheries (Bethesda)* (2009).

[24] Eary LEE (1999). Geochemical and equilibrium trends in mine pit lakes. Appl. Geochemistry.

[25] Yudovich YE, Ketris MP (2006). Selenium in coal: a review. Int. J. Coal Geol..

[26] Boehrer, B. & Schultze, M. Stratification of lakes. *Rev. Geophys*. 1–27 doi:10.1029/2006RG000210.1.INTRODUCTION (2008).

[27] Fisher TSR, Lawrence GA (2006). Treatment of acid rock drainage in a meromictic mine pit lake. J. Environ. Eng..

[28] Fisher, T. S. R. *Limnology of the meromictic island copper mine pit lake.* (University of British Columbia, 2002).

[29] Schindler DW (1980). The effect of fertilization with phosphorus and nitrogen versus phosphorus alone on eutrophication of experimental lakes. Limnol. Oceanogr..

[30] Mueller B, Bryant LD, Matzinger A, Wueest A (2012). Hypolimnetic oxygen depletion in eutrophic lakes. Environ. Sci. Technol..

[31] CCME. *Canadian water quality for the protection of aquatic life - selenium. Canadian Council of Ministers of the Environment* (1999).

[32] Alberta ESRD & Alberta Environment & Parks. *Environmental quality guidelines for Alberta surface Waters*. *Water Policy Branch, Policy Division* doi:10.1002/2014GB005021 (2014).

[33] Goulet R (2001). Test of the first-order removal model for metal retention in a young constructed wetland. Ecol. Eng..

[34] Steinberg N, Blum J, Hochstein L, Oremland R (1992). Nitrate is a preferred electron acceptor for growth of freshwater selenate-respiring bacteria. Appl. Environ. Microbiol..

[35] DeMoll-Decker H, Macy JM (1993). The periplasmic nitrite reductase of Thauera selenatis may catalyze the reduction of selenite to elemental selenium. Arch. Microbiol..

[36] Narasingarao P, Häggblom MM (2007). Identification of anaerobic selenate-respiring bacteria from aquatic sediments. Appl. Environ. Microbiol..

[37] Hockin SL, Gadd GM (2003). Linked Redox Precipitation of Sulfur and Selenium under Anaerobic Conditions by Sulfate-Reducing Bacterial Biofilms. Appl. Environ. Microbiol..

[38] Kumar NR, McCullough CD, Lund Ma, Newport M (2011). Sourcing organic materials for pit lake bioremediation in remote mining regions. Mine Water Environ..

[39] Harrington, J. G. *In situ* immobilization within density variant bodies of water. 12 (2002).

[40] Dessouki TCE, Hudson JJ, Neal BR, Bogard MJ (2005). The effects of phosphorus additions on the sedimentation of contaminants in a uranium mine pit-lake. Water Res..

[41] Lessmann D, Fyson A, Nixdorf B (2003). Experimental eutrophication of a shallow acidic mining lake and effects on the phytoplankton. Hydrobiologia.

[42] McCullough CD, Lund MA, May JM (2008). Field-scale demonstration of the potential for sewage to remediate acidic mine waters. Mine Water Environ..

[43] Subedi G, Taylor J, Hatam I, Baldwin SA (2017). Simultaneous selenate reduction and denitrification by a consortium of enriched mine site bacteria. Chemosphere.

[44] Kuchapski KA, Rasmussen JB (2015). Surface coal mining influences on macroinvertebrate assemblages in streams of the Canadian Rocky Mountains. Environ. Toxicol. Chem..

[45] ALS Environmental. ALS Environmental Laboratory Accreditation. (2016). Available at: http://www.alsglobal.com/Our-Services/Life-Sciences/Environmental/Downloads/North-America-Downloads.

[46] Wallschlager D, Roehl R (2001). Determination of inorganic selenium speciation in waters by ion chromatography-inductively coupled plasma-mass spectrometry using eluant elimination with a membrane suppressor. J. Anal. At. Spectrom..

[47] Wallschlager D, London J (2004). Determination of inorganic selenium species in rain and sea waters by anion exchange chromatography-hydride generation-inductively-coupled plasma-dynamic reaction cell-mass spectrometry (AEC-HG-ICP-DRC-MS). J. Anal. At. Spectrom..

[48] R Core Team. R: A language and environment for statistical computing. http://www.r-project.org/ (2013).

[49] Wickham, H. *ggplot2: elegant graphics for data analysis*, doi:978-0-387-98140-6., (Springer, 2009).

[50] Sarkar, D. *Lattice: multivariate data visualization with R*. (Springer New York, 2008).

