# Supplementary material for: N-P Fertilization Stimulates Anaerobic Selenium Reduction in an End-Pit Lake
Source: Sci Rep. 2017 Sep 5;7:10502. doi: 10.1038/s41598-017-11095-2 (PMC5585328; doi:10.1038/s41598-017-11095-2)
Supplement: Supplementary file 1 — Supplementary Information [file 41598_2017_11095_MOESM1_ESM.pdf]

N-P Fertilization Stimulates Anaerobic Selenium Reduction in an End-Pit Lake

Authors: Andreas Luek*[1], David J. Rowan[2], Joseph B. Rasmussen[1]

Supplementary Materials:

Table S1: Water chemistry summarized by years. Sample depths were at 0.5m below surface (Top) and 1m above bottom (Bottom).

	Year	2012				2013				2014				2015				2016			
		Top		Bottom		Top		Bottom		Top		Bottom		Top		Bottom		Top		Bottom	
Analyte	units	mean	SD	mean	SD	mean	SD	mean	SD	mean	SD	mean	SD	mean	SD	mean	SD	mean	SD	mean	SD
Conductivity	µS/cm	1336	53	1454	62	1313	93	1424	51	1288	122	1455	39	1319	59	1421	30	1275	52	1395	84
Hardness	mg/l	147.5	17.0	176.9	44.0	172.9	48.1	179.9	44.0	153.3	30.9	195.6	52.9	160.6	27.0	175.5	28.4	161.5	39.8	197.3	52.3
pH		8.77	0.23	8.50	0.16	8.57	0.16	8.33	0.18	8.67	0.28	8.39	0.17	8.80	0.16	8.60	0.25	8.74	0.31	8.32	0.19
TDS	mg/l	829.3	34.5	909.8	46.8	852.0	63.4	923.3	27.0	808.0	80.5	921.9	44.2	804.5	40.2	865.6	27.8	782.0	35.1	867.8	37.3
Nitrate	mg/l	0.09	0.04	0.18	0.17	0.27	0.31	1.39	1.74	0.23	0.54	1.89	1.79	0.25	0.21	0.34	0.34	0.22	0.22	0.25	0.44
Ammonia	mg/l	0.12	0.26	0.25	0.47	0.15	0.28	1.23	1.65	0.01	0.01	3.81	3.55	0.05	0.07	3.49	3.69	0.07	0.00	1.27	1.52
Phosphorus	mg/l	0.49	0.64	0.42	0.70	0.10	0.19	0.44	0.36	0.04	0.08	0.45	0.28	0.02	0.02	0.36	0.27	0.04	0.05	0.25	0.20

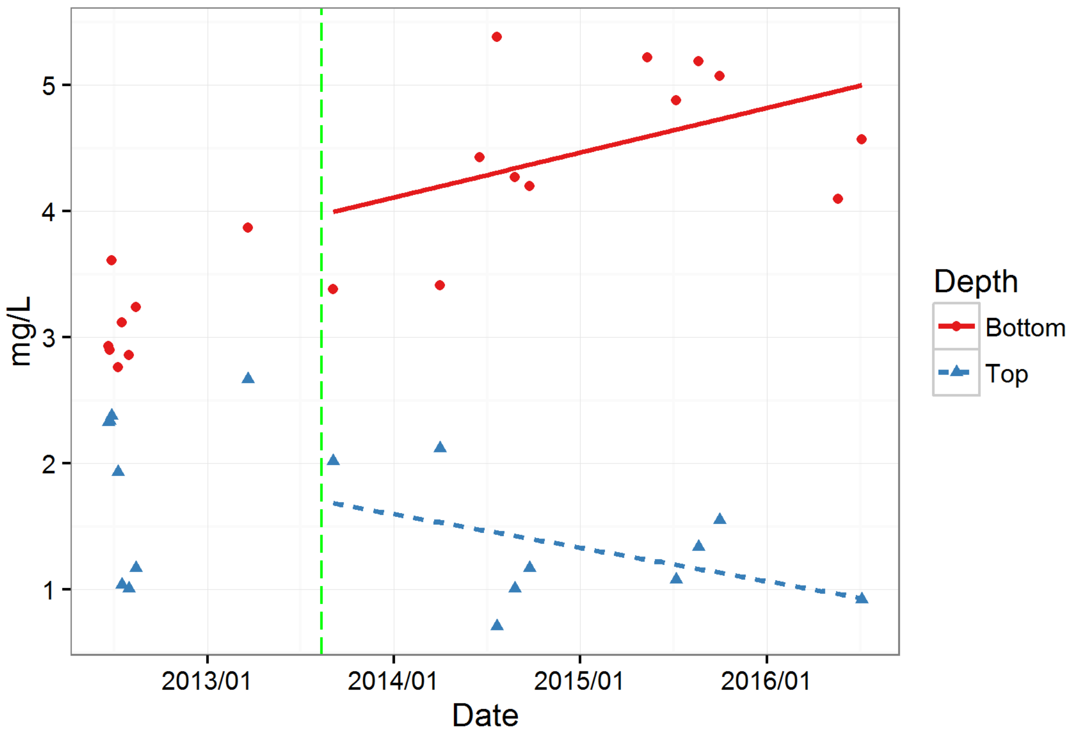

Figure S1: Change in silica concentration over time, indicating an uptake by diatoms at the surface (diminishing concentrations at the surface/blue triangles) and a parallel increase in silica in the bottom samples, due to sinking of decaying diatoms (red circles)
